# Liquid Metal Electrodes: Material Properties, Interfacial Behavior, Fabrication, Performance, and Applications

**DOI:** 10.3390/mi17091018

**Published:** 2026-08-27

**Authors:** Guirong Wu, Gan Wang, Jian Han, Zhichun Liu, Cheng Chen, Rui Li, Chuntao Li, Wei Li, Libo Gao

**Affiliations:** 1 School of Intelligent Manufacturing, Xiamen University, Xiamen 361102, China; wuguirong@stu.xmu.edu.cn (G.W.); han456jian@sina.com (J.H.); 2 Shenzhen Research Institute of Xiamen University, Xiamen University, Shenzhen 518000, China; 3 School of Aerospace Engineering, Xiamen University, Xiamen 361102, China; ganwang@stu.xmu.edu.cn (G.W.); liuzhichun@xmu.edu.cn (Z.L.); chencheng@stu.xmu.edu.cn (C.C.); lirui2022@stu.xmu.edu.cn (R.L.); 4 National Key Laboratory of Aerospace Chemical Power, Xiangyang 441022, China; lichuntao@casc42.cn

**Keywords:** liquid metal electrodes, native oxide layer, flexible electronics, self-healing properties

## Abstract

Liquid metal electrodes (LMEs) combine metallic conductivity with fluid deformability, offering unique advantages for flexible electronics, sensing, biointerfaces, and energy devices. This review examines LMEs from an electrode-centered perspective, linking material composition and oxide-mediated interfacial behavior with fabrication, electrode architecture, performance, reliability, and application readiness. This review further highlights how electrode design and interfacial engineering govern practical electrical and mechanical performance. Emphasis is placed on gallium-based alloys and the dual role of their native oxide layer, which facilitates wetting, adhesion, and pattern stability but may also increase contact resistance and interfacial instability. Representative fabrication strategies are compared in terms of resolution, scalability, adhesion, and deformability, while electrode performance is benchmarked using conductivity or resistance, strain tolerance, cyclic reliability, electrical drift, and activation or self-healing requirements. Applications in flexible electronics, sensors, biomedical interfaces, and energy devices are discussed together with their technological maturity. No single fabrication route or electrode architecture is universally optimal, emphasizing the need for application-specific evaluation. Finally, key challenges in reproducible manufacturing, oxide regulation, contact degradation, leakage prevention, environmental durability, and lifecycle management are highlighted.

## 1. Introduction

Liquid metal electrodes (LMEs) have emerged as an important class of electrically functional soft materials for deformable electronics, energy devices, and biointegrated systems [1,2,3]. The field developed from liquid alloy-filled elastomeric microchannels and mechanically tunable fluidic conductors toward increasingly integrated stretchable circuits and electrodes [4,5]. This evolution is underpinned by the unusual combination of metallic charge transport, liquid-state geometric compliance, and a chemically active surface, features that distinguish gallium-based liquid metals from conventional solid conductors and many conductive composites [6,7]. Gallium-based alloys—especially eutectic gallium–indium (EGaIn) and Ga–In–Sn formulations—are consequently widely used because they remain liquid near room temperature while supporting large deformation, reconfiguration, and damage-tolerant electrical pathways [8,9,10,11].

**Figure 1:**
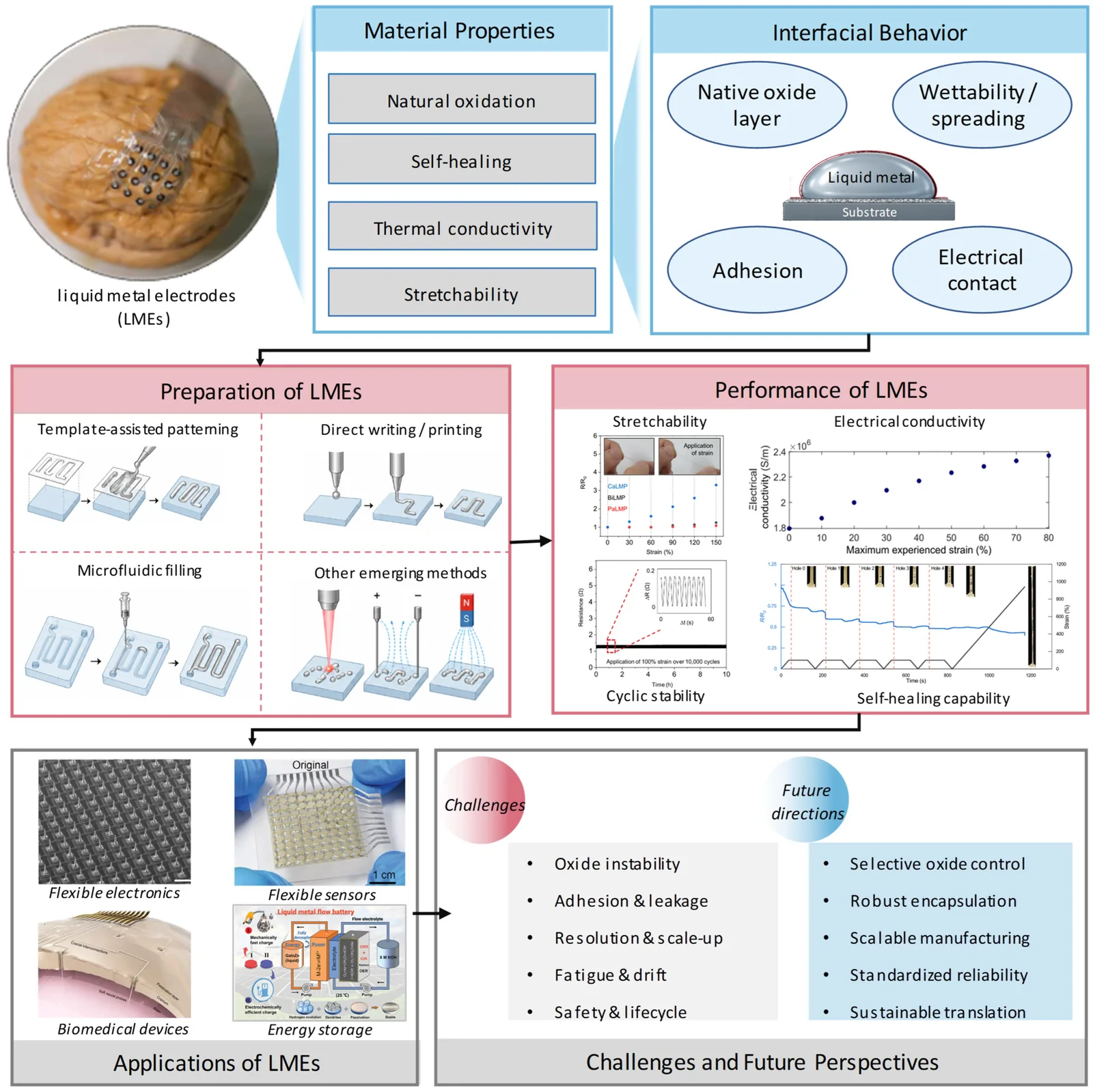
Graphical roadmap of liquid metal electrodes, linking material properties and interfacial behavior with fabrication strategies, performance, representative applications, and future challenges. The overview electrode photograph was reproduced with permission from the American Association for the Advancement of Science (2022) [32]. The stretchability, electrical conductivity, cyclic reliability, and self-healing examples were reproduced with permission from Springer Nature (2023) [33], Springer Nature (2022) [34], Springer Nature (2022) [35], and Springer Nature (2021) [36], respectively. The flexible electronics, flexible sensor, biomedical device, and energy storage application images were reproduced with permission from Springer Nature (2024) [37], Wiley-VCH (2025) [38], the American Association for the Advancement of Science (2022) [32], and Wiley-VCH (2025) [39], respectively.

As summarized in Figure 1, LMEs should be understood as an electrode system rather than simply as a bulk liquid conductor. Their measured behavior reflects alloy composition, confinement, oxide state, conductor geometry, substrate chemistry, packaging, and the nature of the electrical interface. Early EGaIn microchannel structures and high-density soft-electronic patterning established key routes for maintaining conductive pathways during deformation [12,13]. Subsequent self-healing liquid metal/elastomer composites, biphasic Ga–In conductors, distributed liquid metal transmission lines, fiber-like conductors, and optically unobtrusive circuits further broadened the available electrode architectures [14,15,16,17,18]. Gallium-based alloys, particularly EGaIn and Galinstan, therefore dominate ambient temperature soft-electronic research not because they possess a single record property but because low melting temperature, metal-like conductivity, interfacial reconfigurability, and compatibility with multiple manufacturing routes can be combined within one platform.

Over the past decade, significant progress has been made in tailoring the physicochemical properties of liquid metals (e.g., surface tension modulation, oxide layer engineering) and developing advanced fabrication strategies (e.g., microfluidic patterning, direct ink writing) to harness their electrode-specific functionalities. These efforts have unlocked transformative applications ranging from stretchable energy storage devices to implantable neural probes [19,20]. However, the transition from laboratory-scale prototypes to practical implementations remains hindered by unresolved challenges, including dynamic interfacial instability during operation, scalability of manufacturing processes, and long-term electrochemical durability under harsh environments [21,22]. Several reviews have summarized liquid metals from the perspectives of composite material design, flexible and wearable sensors, alloy-specific properties, printing technologies, and flexible electronic devices [22,23,24,25,26]. These studies have established an important foundation for understanding liquid metal materials and their applications. However, most existing reviews are primarily organized according to material categories, fabrication methods, or individual application fields. As summarized in Table 1, oxide-mediated interfacial behavior, electrode architecture, manufacturing trade-offs, condition-aware performance assessment, reliability, and translational maturity have not yet been systematically integrated within a unified electrode-centered framework.

Recent reviews published in 2025–2026 further demonstrate the rapid expansion of the field. Shin et al. summarized gallium-based liquid metal properties, patterning strategies, and soft-electronic applications [27], whereas Zhang et al. provided a broad overview from fundamental properties and droplet engineering to flexible electronic applications [28]. Kim et al. focused specifically on engineering design rules for gallium-based liquid metal bioelectronics [29], Yang et al. reviewed liquid metal particle composites for soft electronics [30], and Wang et al. emphasized materials and architectural design strategies for soft electronic systems [31]. These timely reviews substantially update the materials and device landscape; however, an electrode-defined framework that simultaneously links oxide-mediated interfaces, electrode architecture, manufacturing trade-offs, condition-aware performance benchmarking, failure mechanisms, and translational maturity remains useful for comparing systems that otherwise employ non-equivalent metrics. The present review is, therefore, positioned as a complementary electrode-centered analysis rather than as a broad catalogue of all liquid metal materials.

**Table 1:** Comparison of the scope and analytical emphasis of representative reviews related to liquid metals and liquid metal electrodes.

Systematic Reliability/Stability Analysis	Quantitative Performance Comparison	Fabrication/Process Analysis	Systematic Oxide/Interface Treatment	Representative Emphasis	Review
—	—	✓	—	Flexible/wearable sensor principles and applications	Ren et al. (2020) [24]
—	—	—	✓	EGaIn properties and multidisciplinary applications	Zhao et al. (2023) [25]
—	—	✓	—	Printability, 3D printing, and encapsulation strategies	Zou et al. (2023) [22]
—	✓	✓	—	Stretchable electrodes and substrate integration	Li et al. (2025) [26]
—	—	✓	✓	Fundamental properties, droplet engineering, and broad applications	Zhang et al. (2026) [28]
—	—	✓	—	Composite/architectural design and scalability–resolution trade-offs	Wang et al. (2026) [31]
✓	✓	✓	✓	Electrode-centered integration of interfaces, fabrication, performance, and reliability	This review

Note: ✓ indicates that the corresponding topic is a principal analytical focus or is systematically analyzed in the review, whereas — indicates that it is not a principal analytical focus, although related content may still be discussed. The comparison reflects the primary scope and organizing perspective of each review.

In contrast to previous reviews that primarily catalogue liquid metal materials, fabrication methods, or application examples, the present review adopts an electrode-centered perspective. It connects the dual role of the native oxide layer with electrode architecture, electrical contact, manufacturing resolution, mechanical reliability, and application-specific performance. Manufacturing strategies are evaluated according to their resolution, throughput, adhesion, deformability, scalability, and integration requirements, while representative electrode systems are compared under their original testing conditions rather than through isolated maximum values. Reliability, failure mechanisms, and technological maturity are further considered to distinguish laboratory-scale demonstrations from systems approaching practical implementation. In this review, a liquid metal electrode is defined as an electrically functional device component in which a liquid metal or liquid metal-derived conductive network directly serves as an electrode, current collector, interconnect, charge injection interface, electrochemical reaction interface, or sensing and stimulation electrode. Systems in which the liquid metal functions exclusively as a thermal conductor, passive phase-change material, structural filler, or non-electrical actuation medium are outside the central scope and are discussed only when the liquid metal simultaneously performs an identifiable electrode or current-carrying function.

The remainder of this review is organized as follows. Section 2 examines the composition-dependent properties and interfacial behavior of liquid metals, with particular emphasis on the dual role and selective regulation of the native oxide layer. Section 3 reviews representative electrode architectures and fabrication strategies, evaluates manufacturing trade-offs among resolution, throughput, adhesion, deformability, and scalability, and provides a condition-aware benchmark of electrical, mechanical, self-healing, and long-term reliability metrics. Section 4 discusses representative applications in flexible electronics, sensing systems, biomedical interfaces, and energy-related devices, while distinguishing electrode functions and levels of technological maturity. Section 5 critically examines the principal failure mechanisms and translational barriers, including oxide-related interfacial instability, leakage, delamination, contact degradation, fatigue-induced resistance drift, manufacturing reproducibility, safety, and lifecycle management. Corresponding future directions are discussed in terms of selective oxide regulation, robust encapsulation, standardized reliability assessment, scalable manufacturing, and sustainable implementation. Finally, Section 6 summarizes the principal conclusions. Figure 1 presents the electrode-centered framework linking interfacial state, electrode architecture, manufacturing, condition-aware performance, reliability, and translational readiness.

## 2. Material Properties and Interfacial Behavior of Liquid Metal Electrodes

### 2.1. Composition, Phase State, and Electrode-Relevant Properties

Liquid metals (LMs) combine metallic electrical conduction with fluid mechanical compliance, but these descriptors are insufficient by themselves for electrode design. Electrical transport, wetting, pattern retention, contact resistance, and deformation stability depend on alloy composition, temperature, oxidation, geometry, and confinement [6,7]. For this reason, the following discussion distinguishes bulk thermophysical properties from effective properties measured in printed traces, particle networks, composites, or microfluidic electrodes.

**Figure 2:**
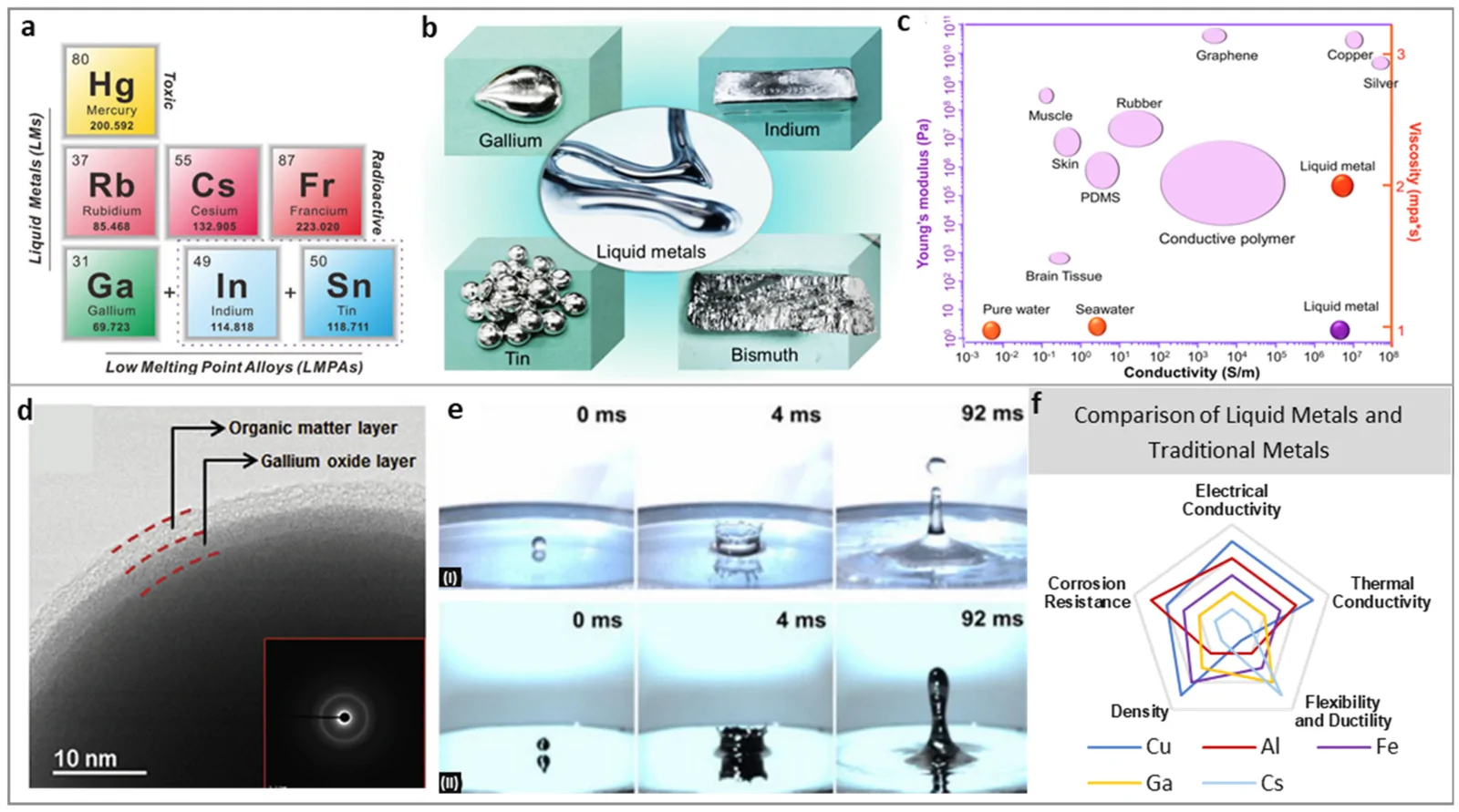
Representative material characteristics of liquid metals. (**a**) Low-melting-point metallic elements. (**b**) Representative low-melting metals and liquid metal systems. (**c**) Materials selection map comparing electrical conductivity, mechanical compliance, and viscosity. (**d**) Nanoscale oxide layer at a liquid metal interface. (**e**) Impact and spreading of aqueous and gallium-based liquid metal droplets. (**f**) Qualitative comparison with conventional metals. Panels (**a**–**e**) were reproduced with permission from the Royal Society of Chemistry (2018) [41], Wiley-VCH (2026) [42], Elsevier (2020) [23], Wiley-VCH (2016) [43], and Elsevier (2014) [47], respectively.

The thermophysical properties of Ga–In–Sn alloys depend strongly on exact composition and operating temperature. For eutectic Ga_67_In_20.5_Sn_12.5_ (wt.%), Plevachuk et al. reported an electrical conductivity of 3.26 × 10^6^ S·m^−1^ at 300 K using a four-point contact method under argon and a dynamic viscosity of 2.09 mPa s at 299 K using an oscillating cup viscometer [40]. These values are, therefore, alloy- and condition-specific rather than universal constants for gallium-based liquid metals. Figure 2a places representative low-melting metallic elements in the periodic table, while Figure 2b illustrates representative low-melting metals and liquid metal systems [41,42].

Low-melting metallic systems include gallium-based alloys, mercury, alkali metal alloys, and indium-, tin-, bismuth-, or antimony-containing fusible alloys. These systems are not interchangeable. Gallium-based alloys dominate ambient temperature deformable electrodes because they combine a near-room-temperature liquid state, metallic conductivity, low volatility, and relatively favorable handling characteristics. In addition, their native oxide layer provides an unusual interfacial functionality that can facilitate wetting, adhesion, and pattern retention, making these alloys particularly compatible with soft and stretchable device fabrication.

However, gallium-based systems are not universally optimal. Their oxide layer can increase contact resistance and introduce interfacial instability, while alloy composition, operating temperature, and interactions with contacting materials may influence phase stability and long-term reliability. Mercury provides a continuously renewable liquid surface and remains useful in specialized electroanalytical interfaces, but its broader use is severely constrained by toxicity. Alkali metal-based liquids can provide high electrochemical activity and are, therefore, attractive for specific electrochemical or energy-related systems, although stringent isolation from oxygen and moisture is required. In/Sn/Bi-based fusible metals are more suitable for applications in which moderate or elevated operating temperatures are acceptable and where a solid–liquid phase transition can be deliberately exploited. Therefore, the predominance of gallium-based liquid metals is application-specific rather than universal, and alloy selection should be matched to the thermal, electrochemical, mechanical, interfacial, and safety requirements of the intended electrode.

Figure 2c compares representative liquid metals with conventional materials in terms of electrical conductivity, mechanical compliance, and viscosity, highlighting the unusual coexistence of metal-like transport and fluid-like deformability [23]. Figure 2d shows a nanoscale oxide/organic interfacial shell surrounding a liquid metal nanodroplet [43]. In oxygen-containing environments, gallium-rich surfaces rapidly form a Ga_2_O_3_-rich skin; this interphase can support finite interfacial stress and strongly modify the apparent surface tension, wetting, adhesion, rheology, pattern retention, and electrical contact behavior [12,44,45,46]. Consequently, values reported for a nominally identical alloy can differ substantially when the oxide state or test environment is changed.

The droplet impact sequences in Figure 2e further illustrate that ambient gallium-based liquid metals cannot always be treated as simple Newtonian liquids [47]. Their bulk viscosity is low, yet oxidation can introduce interfacial elasticity, pinning, and non-equilibrium shape retention; oxidation-mediated fingering and related instabilities provide particularly clear examples of how surface chemistry can reshape macroscopic flow [48]. Apparent spreading and recoil, therefore, reflect coupled inertia, surface tension, wettability, viscous dissipation, and oxide-mediated interfacial stress rather than a single intrinsic viscosity value. Figure 2f provides a qualitative comparison of the overall performance characteristics of liquid metals and conventional solid metals. While conventional metals generally exhibit superior intrinsic electrical conductivity and mechanical robustness, liquid metals offer distinct advantages in deformability, stretchability, self-healing capability, and conformability, enabling them to maintain electrical continuity under large and repeated mechanical deformation. These characteristics make liquid metals particularly attractive for soft, stretchable, and dynamically reconfigurable electrodes. Nevertheless, their practical performance is strongly governed by interfacial phenomena, including oxide formation, wetting and adhesion, substrate compatibility, and encapsulation. Therefore, understanding and regulating the liquid metal/substrate interface is essential for translating these intrinsic material advantages into reliable electrode performance, as discussed in Section 2.2.

### 2.2. Dual Role of the Native Oxide Layer

**Figure 3:**
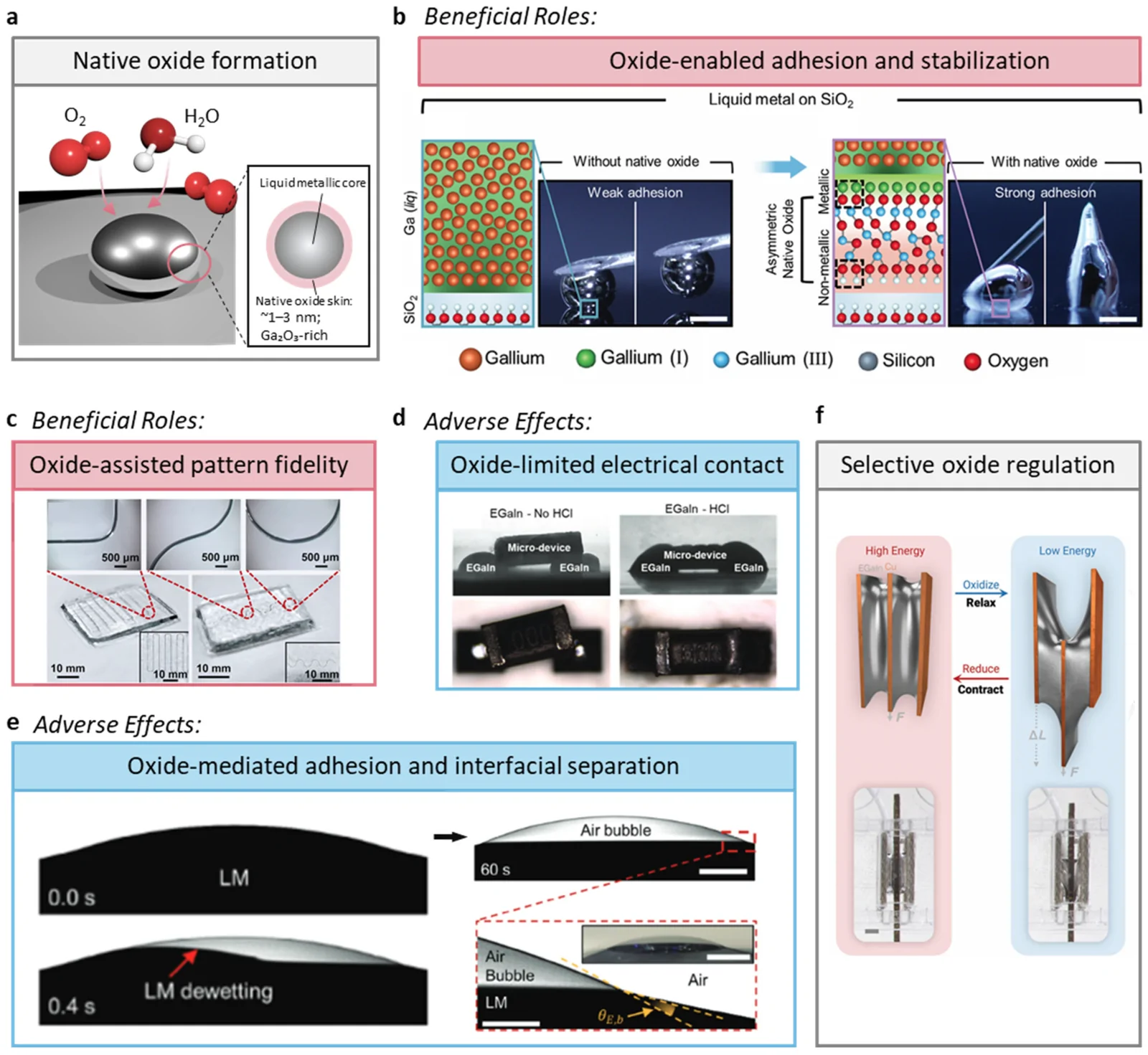
Dual role and regulation of the native oxide layer in gallium-based liquid metals. (**a**) Native oxide formation. (**b**) Oxide-enabled adhesion and stabilization. (**c**) Oxide-assisted pattern fidelity. (**d**) Oxide-limited electrical contact. (**e**) Oxide-mediated adhesion and interfacial separation. (**f**) Selective oxide regulation. Panels (**b**–**d**,**f**) were reproduced with permission from Wiley-VCH (2024) [50], Springer Nature (2022) [51], Wiley-VCH (2018) [52], and Wiley-VCH (2022) [53], respectively.

Gallium-based liquid metals spontaneously develop a nanometer-scale, Ga_2_O_3_-rich oxide skin in oxygen-containing environments. The surface can switch from liquid-like capillary behavior to a mechanically active interface as oxidation proceeds, and electrochemical control can produce correspondingly large changes in surface activity [12,44,45,46,49]. This dynamic interphase repeatedly ruptures and reforms during printing, deformation, particle activation, and electrochemical operation; it, therefore, influences rheology, wetting, adhesion, electrical contact, and long-term stability simultaneously. Figure 3a summarizes native oxide formation and establishes the basis for the beneficial, adverse, and actively regulated behaviors shown in Figure 3b–f.

Native oxide can play a constructive role when interfacial adhesion and geometric stabilization are required. As shown in Figure 3b, Jung et al. demonstrated that the spontaneously formed 2–3 nm gallium oxide layer is chemically asymmetric rather than a simple passive shell. The oxide surface facing the liquid metal contains suboxide species with Ga-rich character, which interact favorably with the underlying liquid metal, whereas the outer surface is predominantly fully oxidized and hydroxyl-terminated, enabling polar interactions with oxide substrates such as SiO_2_. This asymmetric interfacial chemistry allows oxide-coated gallium to adhere to SiO_2_, whereas oxide-free gallium shows negligible wetting on the same hydroxylated surface. More importantly, Johnson–Kendall–Roberts measurements revealed an exceptionally low liquid metal/interior oxide interfacial energy of 9.8 ± 1.4 mN·m^−1^, compared with approximately 724 mN·m^−1^ for the bare metal interface, explaining why the oxide can stabilize non-spherical liquid metal configurations [50].

A related mechanical stabilization effect is exploited during extrusion printing, as illustrated in Figure 3c. Wu et al. dispersed Galinstan into an acrylamide/nanoclay yield-stress support bath containing H_2_O_2_, which rapidly oxidized the freshly extruded metal surface. Without H_2_O_2_, the high interfacial tension caused the extrudate to break into nearly spherical droplets, while 0.67% H_2_O_2_ produced only partially stabilized, tadpole-like segments. At 1.33% H_2_O_2_, rapid formation of the oxide skin was sufficient to resist capillary-driven breakup, allowing continuous and uniform liquid metal filaments with typical diameters of approximately 120–190 μm and enabling complex suspended three-dimensional structures. Increasing the H_2_O_2_ concentration further to 2.00% produced no additional improvement, indicating that oxidation is beneficial only once an appropriate interfacial state is established rather than through unrestricted oxide growth [51].

Together, these studies show that the positive role of the native oxide arises from two distinct mechanisms: chemically asymmetric interfacial bonding that promotes adhesion, and mechanically robust interfacial skin formation that counteracts capillary instability. Thus, oxide should be regarded as an actively tunable interfacial component rather than simply an unavoidable surface contaminant.

The same oxide becomes detrimental when low-resistance electrical contact or reversible interfacial motion is required. As shown in Figure 3d, the native oxide introduces an electrically resistive barrier at EGaIn–metal interfaces, leading to unstable and poorly reproducible soft–rigid electrical contacts. HCl-assisted oxide removal exposes the metallic surface and markedly improves direct electrical coupling, highlighting the importance of oxide control for reliable interconnection [52]. A different failure mode is illustrated in Figure 3e. Because oxide can adhere strongly to the surrounding substrate while the liquid metal maintains a comparatively low interfacial energy with the inner oxide surface, mechanical deformation may cause the liquid metal to retract or separate from its oxide envelope, producing local dewetting and loss of interfacial continuity [50]. These observations indicate that oxide-assisted adhesion, low contact resistance, and reversible interfacial motion are not inherently compatible and, therefore, must be balanced according to the specific electrode architecture and operating conditions.

Selective oxide regulation is, therefore, more useful than either complete preservation or indiscriminate removal. Electrochemical oxidation and reduction can reversibly change the liquid metal surface state and associated interfacial tension, producing controllable changes in shape and mechanical response (Figure 3f) [53,54]. For reproducible LMEs, studies should report atmosphere or electrolyte composition, pH, applied potential and reference electrode where relevant, oxide removal or activation procedure, contact resistance, and cyclic interfacial stability. Oxide should be treated as a process variable that connects pattern fidelity and wetting with electrical reliability rather than as an uncontrolled by-product.

At the particle scale, oxide regulation becomes a shell engineering and activation problem rather than only a bulk interface problem. Flow focusing, ultrasonication, and other dispersion routes can generate EGaIn or gallium-based droplets whose stability is governed by oxide formation and surface chemistry [55,56]. Thiolation and other ligand treatments can deliberately modify oxide growth and particle surfaces [57], while mechanical rupture or sintering can break the electrically resistive shell and recover conductive interparticle pathways [58,59]. Phase separation and composition redistribution within liquid metal nanoparticles further demonstrate that nanoscale particles are not simply miniaturized copies of the bulk alloy [60]. Broad particle-focused studies have accordingly emphasized the coupled roles of particle size, shell chemistry, activation, and matrix interaction [61]. Recent carbon encapsulation and electroactive ligand strategies extend this concept by improving particle confinement or dispersion [62,63], but any gain in storage or environmental stability must be assessed together with conductivity after activation and contact persistence during cyclic deformation.

## 3. Fabrication and Performance of Liquid Metal Electrodes

Liquid metal electrode fabrication must preserve metallic conduction while controlling a fluid conductor that is highly sensitive to oxide state, wetting, confinement, and substrate chemistry. Consequently, fabrication quality cannot be judged only by whether a conductive pattern is obtained; feature fidelity, electrical activation, adhesion, leakage resistance, deformation tolerance, throughput, and compatibility with encapsulation and component integration must be evaluated together.

The major fabrication routes considered here are template-assisted patterning, direct writing/printing, microfluidic or capillary confinement, and emerging field-, electrochemical-, or surface-assisted methods. Each route solves a different combination of geometric and interfacial constraints, and none simultaneously maximizes resolution, throughput, conductivity, deformability, and reliability.

Section 3.1, therefore, compares representative fabrication strategies and their application windows, while Section 3.2 evaluates performance under the original test conditions reported for each architecture. This separation is important because extreme strain, high conductivity, small feature size, and long cycling life are often demonstrated using different material states and different device geometries.

### 3.1. Fabrication Strategies

#### 3.1.1. Template-Assisted Patterning

**Figure 4:**
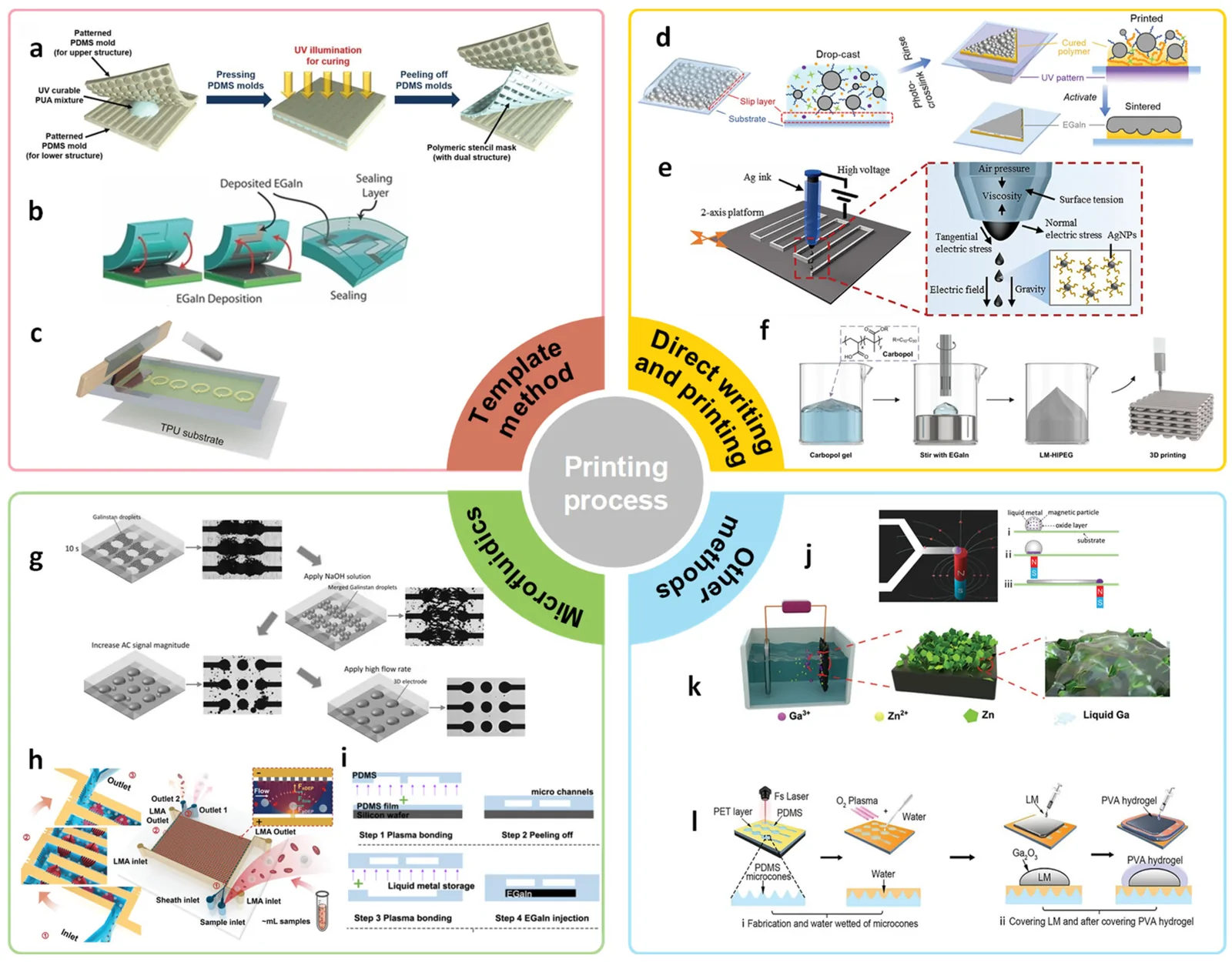
Representative fabrication strategies for liquid metal electrodes. (**a**–**c**) Template-assisted and screen-printing approaches. (**d**–**f**) Direct writing and printing. (**g**–**i**) Dielectrophoretic and microfluidic/capillary patterning. In panel (**h**), numbers 1–3 denote the sequential stages of particle separation: (1) sample introduction and focusing, (2) progressive DEP-induced deflection, and (3) collection of separated particles at the outlets. (**j**–**l**) Magnetic field, electrochemical interface, and surface-assisted methods. Panels (**a**–**l**) were reproduced with permission from Wiley-VCH (2020) [64], Wiley-VCH (2014) [13], Springer Nature (2025) [68], Wiley-VCH (2024) [74], Wiley-VCH (2024) [75], Springer Nature (2024) [76], Wiley-VCH (2015) [90], Wiley-VCH (2024) [91], Wiley-VCH (2019) [92], Wiley-VCH (2025) [93], and Wiley-VCH (2025) [38], respectively.

Template-assisted patterning remains attractive for planar LMEs because a predefined mask, stencil, or mold can establish many features in one deposition step. Park et al. used a dual-structured polymer template to define high-fidelity EGaIn patterns (Figure 4a) [64], while Gozen et al. demonstrated high-density soft-matter electronic features with micron-scale line widths (Figure 4b) [13]. Transfer printing and dual-transfer strategies similarly exploit controlled wetting and release to move liquid metal features between substrates [65,66], and stencil-based patterning provides another route to parallel, repeatable conductor formation [67]. More recently, fully screen-printed stretchable multilayer liquid metal circuits have been produced using scalable printing and water spray sintering (Figure 4c) [68].

The main advantage of template, stencil, transfer, and screen printing routes is parallel planar pattern definition with relatively simple tooling. Large-area conductor fabrication has also been demonstrated on diverse surfaces, emphasizing the manufacturing potential of interface-controlled deposition [69]. Their limitations arise from mask registration, wetting and edge retraction, transfer or peel-off fidelity, residue, and delamination under strain. Practical resolution is, therefore, set not only by nominal mask dimensions but also by the liquid metal/substrate interfacial state and any post-patterning activation step.

For scale-up, template-based studies should report line-width distributions, yield across repeated devices, sheet or line resistance variation, alignment tolerance for multilayer structures, and performance after encapsulation rather than presenting only the smallest successful feature.

#### 3.1.2. Direct Writing and Printing

Direct writing and printing provide greater geometric freedom and are well suited to customized patterns, non-planar substrates, and multimaterial integration. Shear-driven direct writing established a route for dispensing room-temperature gallium-based alloys while controlling filament formation [70,71], and free-standing as well as high-resolution three-dimensional liquid metal structures have subsequently been demonstrated through oxide-assisted and reconfigurable printing strategies [72,73]. Projection lithography-based liquid metal printing enables rapid fabrication of fine, highly deformable circuits (Figure 4d) [74], while microconfined assembly provides another high-resolution route to mechanically robust stretchable electrodes (Figure 4e) [75]. In a complementary ink design strategy, EGaIn dispersed in a Carbopol-based high-internal-phase emulsion gel can be directly written as a three-dimensional ink and subsequently activated to establish electrical conduction (Figure 4f) [76].

These examples illustrate the central trade-off within direct printing: bulk-liquid metal routes retain high intrinsic conductivity but remain sensitive to wetting, oxidation, and line breakup, whereas particle, aerosol, or emulsion inks improve printability and structural stability at the cost of additional activation or percolation steps. Aerosol deposition, photonic sintering, evaporation-induced sintering, and colloidal self-assembly/microtransfer printing illustrate the breadth of activation concepts now used to convert printable liquid metal particles into conductive features [77,78,79,80]. Accordingly, reported resolution should be interpreted together with particle state, post-processing sequence, conductivity after activation, and the electrical uniformity of repeated features.

Integration with conventional metals and components introduces a second manufacturing problem: the interface must function as a mechanically compliant solder or contact rather than merely as a printed trace. Room-temperature EGaIn-assisted sintering of silver nanoparticles, molecularly modulated soldering, stretchable liquid metal solder stickers, and intermetallic wetting strategies demonstrate different approaches to reducing contact resistance and stabilizing heterogeneous interfaces [81,82,83,84]. Future direct-printing platforms should, therefore, report not only line resolution but also contact resistance, alignment yield, soft–rigid fatigue, activation burden, and electrical variation across large areas.

Scalability is increasingly being addressed through wafer-scale transfer, interface fusion approaches, micromesh architectures, quantitative spray processing, and fully screen-printed multilayer circuits [68,85,86,87,88]. These studies reinforce that manufacturing readiness should be evaluated by areal throughput, registration accuracy, post-processing burden, device-to-device electrical variation, and long-term reliability rather than by minimum linewidth alone.

#### 3.1.3. Microfluidic Technology

Microfluidic and capillary strategies exploit geometric confinement or surface force-controlled transport to position liquid metal. Vacuum-assisted filling has been shown to improve access to complex microchannel networks [89], complementing earlier EGaIn microchannel platforms [12]. Tang et al. used dielectrophoresis to create three-dimensional Galinstan microstructures for enhanced particle manipulation (Figure 4g) [90], while Chai et al. developed capillarity-enabled large-array liquid metal electrodes for dielectrophoretic microfluidics, with capillary control supporting both array formation and channel integration (Figure 4h,i) [91].

Confinement provides strong containment and large deformability, but its manufacturing burden shifts to channel fabrication, filling reliability, trapped gas, ports, bonding interfaces, and multilayer alignment. Surface oxidation can also alter flow and filling behavior, and low-voltage electrochemical control has been used to steer liquid metal within microchannels [54]. The nominal channel dimension, therefore, does not by itself describe device yield, leakage resistance, or electrical uniformity.

Capillary and passive flow designs can reduce external pumping and simplify large-array filling, yet the acceptable process window remains sensitive to channel geometry, surface energy, and liquid metal oxidation. For high-throughput microfluidic LMEs, filling completeness and electrical uniformity across the full array should be reported statistically.

Microfluidic fabrication is thus particularly valuable when containment and mechanical compliance dominate the design requirements, whereas open-surface printing is preferable when rapid access, reconfiguration, or direct component attachment is required.

#### 3.1.4. Other Methods

Other emerging fabrication strategies exploit external fields, electrochemical reactions, or engineered surface microstructures to manipulate liquid metals beyond conventional printing. In magnetic field-assisted patterning, magnetic Ni microparticles are incorporated into the liquid metal to impart remote magnetic responsiveness. A representative formulation containing approximately 3.3 wt% Ni enables the liquid metal to be guided and patterned not only on planar substrates but also on curved or poorly wettable surfaces, providing greater geometric freedom than conventional nozzle-based deposition (Figure 4j) [92]. Electrochemical processing offers a different route by generating a functional liquid metal-containing interface directly during metal deposition. In situ Zn/Ga co-deposition produces a liquid Ga-rich interfacial phase that modifies Zn nucleation, redistributes the local electric field and ion flux, and consequently promotes more uniform Zn growth under relatively high deposition current densities (Figure 4k) [93]. Surface engineering provides another complementary strategy. Femtosecond laser-generated microstructures, combined with water/hydrogel-assisted wetting, can spatially confine liquid metal and form embedded microstructured electrodes with enhanced interfacial adhesion and reduced mechanical coupling between neighboring units (Figure 4l) [38].

Together, these approaches extend liquid metal fabrication from simple geometric patterning toward active regulation of magnetic forces, electrochemical nucleation, wetting, and interfacial confinement. However, their broader application will depend on whether magnetic particle incorporation, electrolyte control, laser processing, and surface pretreatment can be simplified for reproducible large-area manufacturing without introducing additional contamination, processing complexity, or long-term interfacial instability.

#### 3.1.5. Fabrication Trade-Offs and Application Windows

Taken together, the strategies in Section 3.1.1, Section 3.1.2, Section 3.1.3 and Section 3.1.4 show that no single process simultaneously maximizes feature resolution, interfacial adhesion, deformability, throughput, scalability, and long-term reliability. Route selection should instead follow the physical state of the liquid metal, the required electrode geometry, substrate chemistry, integration sequence, and operating environment. Oxide state is a cross-cutting process variable: it can assist shape retention and adhesion in bulk liquid metal patterning but can impede particle-to-particle and soft–rigid electrical contact.

Template-assisted patterning is well suited to repeated planar structures and batch definition, whereas direct writing offers higher geometric freedom and easier customization. Particle and composite inks improve processability and containment but commonly introduce activation or percolation requirements. These distinctions mean that a nominal linewidth should never be reported without the associated electrical metric, post-processing state, and deformation condition.

Microfluidic confinement is advantageous when leakage prevention and large deformation are priorities, but channels, ports, filling completeness, and bonding interfaces become critical failure points. Field-, electrochemical-, and surface-assisted methods can provide localized manipulation or specialized interfaces, yet their equipment dependence and process complexity may limit throughput or substrate generality.

Accordingly, Table 2 compares fabrication strategies in terms of practical process windows rather than ranking them by a single metric. Meaningful manufacturing comparisons should include resolution distribution, throughput, yield, electrical uniformity after processing, adhesion, cyclic resistance drift, leakage resistance, environmental durability, and compatibility with system-level assembly.

### 3.2. Performance Benchmarking of Liquid Metal Electrodes

In recent years, liquid metal has been extensively researched and developed as an excellent electrode material. A quantitative comparison of liquid metal electrodes requires the material state, electrode architecture, substrate or matrix, encapsulation, conductor geometry, deformation mode, and electrical measurement method to be considered together. The bulk conductivity of a continuous liquid alloy, the effective conductivity of an activated particle network, the sheet resistance of a patterned film, the contact resistance of a soft–rigid junction, and the impedance of a bioelectrode describe different physical quantities and should not be treated as interchangeable. Table 3, therefore, summarizes representative systems under their original testing conditions. Values are included only when they are explicitly reported in the primary study, whereas NR is used when a parameter is unavailable.

Electrical performance and fabrication architecture. Electrical performance should be evaluated together with the electrode structure and measurement method. Projection-patterned and electrohydrodynamically confined electrodes can achieve microscale features while maintaining conductivities of about 10^6^ S·m^−1^. For multilayer and composite electrodes, the reported conductivity may also include contributions from solid metals or conductive particle networks. Therefore, Table 3 keeps the original material state and test conditions for each value. Conductivity, sheet resistance, contact resistance, and bioelectrode impedance are listed separately because they describe different electrical properties.

**Figure 5:**
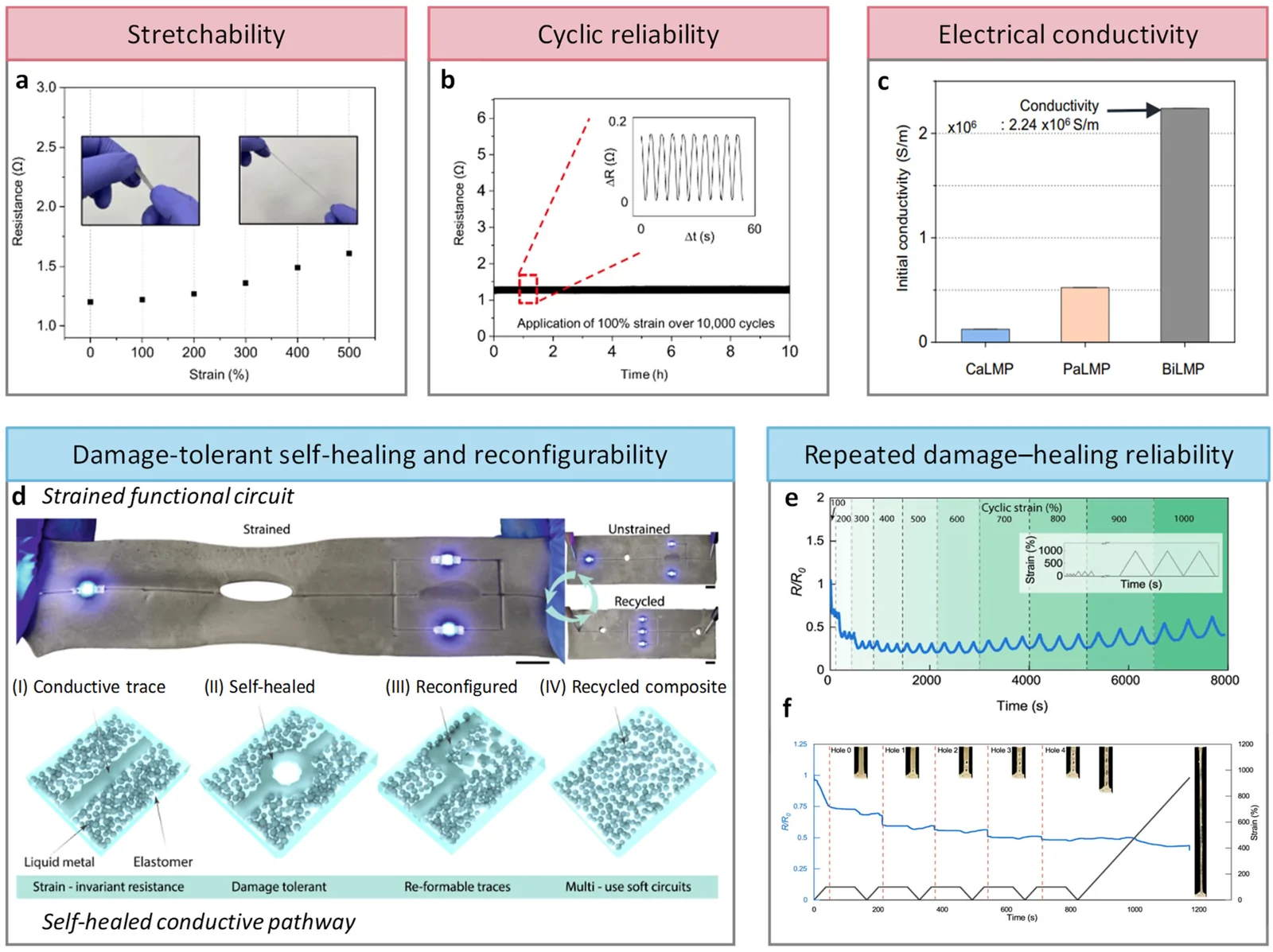
Representative performance and reliability characteristics of liquid metal electrodes. (**a**) Stretchability. (**b**) Cyclic reliability. (**c**) Electrical conductivity. (**d**) Damage-tolerant self-healing and reconfigurability. (**e**,**f**) Repeated damage healing reliability. Panels (**a**–**f**) were reproduced with permission from Springer Nature (2022) [35], Springer Nature (2023) [33], and Springer Nature (2021) [36], respectively.

Stretchability and cyclic reliability are likewise distinct metrics. In the semi-solid PaLMP conductor shown in Figure 5a, resistance changes only slightly during stretching; the original study reports an increase of approximately 0.1 Ω at 200% strain [35]. Figure 5b shows repeated application of 100% strain over 10,000 cycles without electrical failure under the reported conditions. These results illustrate why a deformation-dependent resistance curve and a defined cyclic protocol are more informative than a maximum strain value alone.

Electrical conductivity should also be separated from strain tolerance. The bilayer EGaIn particle composite in Figure 5c reaches an initial conductivity of 2.24 × 10^6^ S·m^−1^, while retaining a packed bilayer architecture designed to improve mechanical durability [33]. Because this value belongs to a specific composite architecture, it should not be interpreted as the intrinsic bulk conductivity of EGaIn. Cross-study comparison, therefore, requires the conductive phase, layer structure, geometry, and activation state to be stated explicitly.

Damage tolerance introduces another performance dimension that cannot be captured by conventional cyclic stretching. The liquid metal/elastomer composite in Figure 5d–f forms conductive traces by reorganizing discrete liquid metal droplets and can self-heal electrically after severe local damage, be locally reconfigured, and be recycled into new circuits [36]. The original study reports stretchability to 1200% strain with minimal resistance change and maintenance of electrical functionality during multiple damage events. These observations should be described as damage-triggered conductive network reconfiguration rather than as recovery of every mechanical property of the polymer matrix.

Taken together, Figure 5 and Table 3 show that conductivity, stretchability, cyclic reliability, self-healing, and reconfigurability represent complementary rather than interchangeable performance axes. Future reports should, therefore, specify the liquid metal composition and physical state, electrode geometry and encapsulation, initial electrical metric, contact resistance where relevant, maximum operational strain with a failure criterion, cyclic amplitude/frequency/number, residual resistance after unloading, damage mode, recovery time, activation requirement, and the thermal or environmental conditions used for ageing.

Terminal contacts and interfaces remain a major source of uncertainty because record conductor performance can be lost at soft–rigid junctions. Reliability assessment should, therefore, include contact resistance evolution, baseline drift, failure localization, and post-test inspection in addition to bulk or line resistance. This interface-aware reporting is particularly important when comparing particle composites, microchannels, multilayer conductors, and integrated bioelectrodes.

## 4. Applications of Liquid Metal Electrodes

Liquid metals, owing to their excellent electrical conductivity, remarkable stretchability, and self-healing capabilities, exhibit significant potential in flexible electronic devices, sensors, biomedical applications, and energy systems. In recent years, these unique properties have not only driven the development of novel materials but also provided new perspectives for innovation in device architectures and functional integration. This chapter systematically examines the representative applications of liquid metal electrodes across these key fields, together with their underlying physical properties and operating mechanisms.

In the realm of flexible electronic devices, the high conductivity and fluidity of liquid metals provide a solid foundation for the realization of stretchable circuits and flexible displays, which are expected to play a pivotal role in next-generation wearable devices and smart display technologies. In sensor applications, the deformation response of liquid metals enables highly sensitive detection of multiple parameters, such as pressure, temperature, and strain, significantly enhancing sensor response speed and stability. Meanwhile, in biomedical applications, liquid metals serve as key materials in biosensors and implantable electrodes, meeting biocompatibility requirements while advancing medical monitoring and neural interface technologies. Finally, in energy systems, liquid metal electrodes serve as flowable anodes, current-carrying interfaces, and deformable electrodes for energy storage and energy conversion devices.

### 4.1. Flexible and Stretchable Electronics

Liquid metals are particularly attractive for flexible and stretchable electronics because they can function as deformable interconnects while preserving metallic electrical transport across bending, stretching, and torsion. Distributed liquid metal transmission lines, sheath core fibers, biphasic conductors, and self-healing composites illustrate several complementary approaches to maintaining conduction during large deformation [14,15,16,17]. The central engineering challenge has, therefore, shifted from demonstrating a stretchable trace to integrating that trace with substrates, rigid components, multilayer routing, encapsulation, and repair strategies without creating dominant soft–rigid failure points.

**Figure 6:**
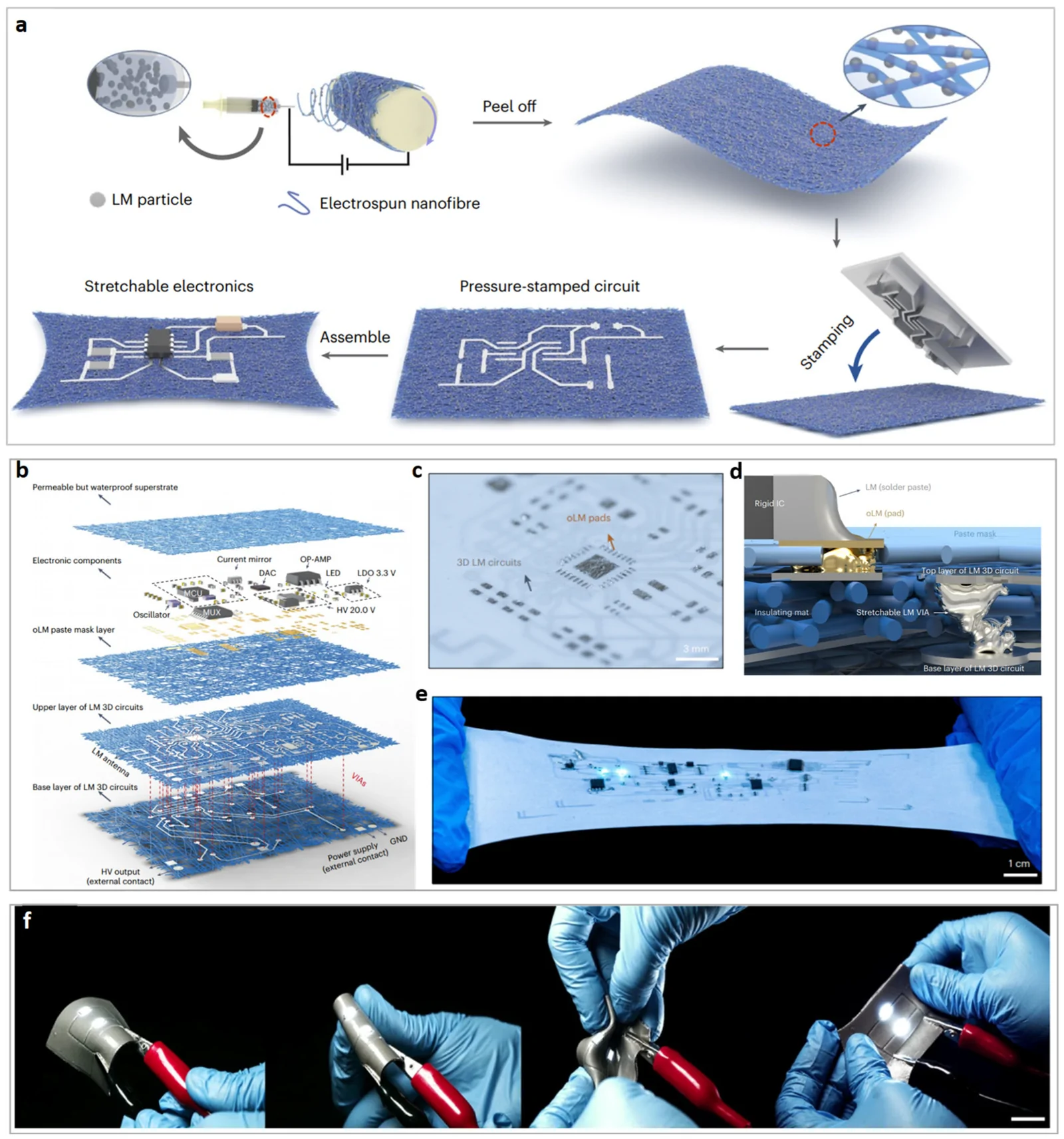
Representative liquid metal-enabled flexible and stretchable electronic systems. (**a**) Pressure-stamped stretchable electronics based on a liquid metal particle nanofiber membrane. (**b**–**e**) Permeable multilayer electronic skin with stretchable hybrid liquid metal solder and soft–rigid interconnects. (**f**) Deformable self-healing liquid metal circuit (the scale bar represents 10 mm). Panels (**a**–**f**) were reproduced with permission from Springer Nature (2024) [98], Springer Nature (2024) [99], and Springer Nature (2021) [36], respectively.

Figure 6a shows a pressure stamping strategy in which a nanofiber membrane containing semi-embedded liquid metal particles is transferred to form stretchable electronic patterns [98]. The approach illustrates how particle confinement and transfer printing can improve pattern handling while retaining deformability.

At a higher level of integration, the permeable electronic skin in Figure 6b–e combines stretchable hybrid liquid metal solder with rigid electronic components and multilayer soft structures [99]. The device architecture highlights a key requirement for practical LMEs: the conductor itself must remain reliable at vias, pads, component interfaces, and encapsulation boundaries, not only along free-standing traces.

Figure 6f provides a complementary material-level strategy based on a self-healing and reconfigurable liquid metal composite [36]. Because conductive pathways can reorganize after local damage, such systems offer a route toward fault-tolerant soft circuits; however, electrical healing should be distinguished from full structural restoration, and repeated repairability must be quantified under application-relevant loading.

Together, these examples show progression from deformable conductors to integrated and damage-tolerant electronic systems. Future advances will depend on interconnect density, assembly yield, component attachment, moisture and abrasion resistance, repairability, and stable electrical behavior under combined stretching, bending, torsion, and repeated handling.

Recent work further indicates that strain decoupling and function-specific signal stability are becoming as important as maximum stretchability. This shift is appropriate because high integration density can make packaging and soft–rigid interfaces the dominant reliability bottlenecks even when the liquid metal conductor itself remains intact.

Flexible electronic demonstrations now show convincing mechanical integration, but practical readiness still depends on manufacturing yield, soft–rigid interconnect fatigue, encapsulation, washability/abrasion resistance, and repeatable repair or replacement at the system level.

### 4.2. Flexible Sensors

Liquid metal sensors can exploit continuous traces, microchannels, particle composites, and compliant capacitive electrodes, but these architectures should not be assigned a single sensing mechanism. Microfluidic pressure sensors, microbump-assisted pressure sensors, torsion/strain/touch sensors based on liquid metal fibers, inertial devices, and multifunctional wearable platforms demonstrate how geometry and mechanical coupling can be tailored to different measurands [100,101,102,103,104]. Continuous conductors are dominated by geometry and contact evolution, whereas particle systems can additionally involve oxide shell rupture, percolation, and local conductive network reconfiguration. This distinction is essential when comparing sensitivity, hysteresis, drift, bandwidth, and durability.

For a continuous liquid metal strain sensor, the resistance is primarily governed by the geometric deformation of the conductive pathway:

(1) $$R\;=\;\frac{\rho \cdot L}{A}$$

where ρ, *L*, and *A* denote the electrical resistivity, conductor length, and cross-sectional area, respectively.

**Figure 7:**
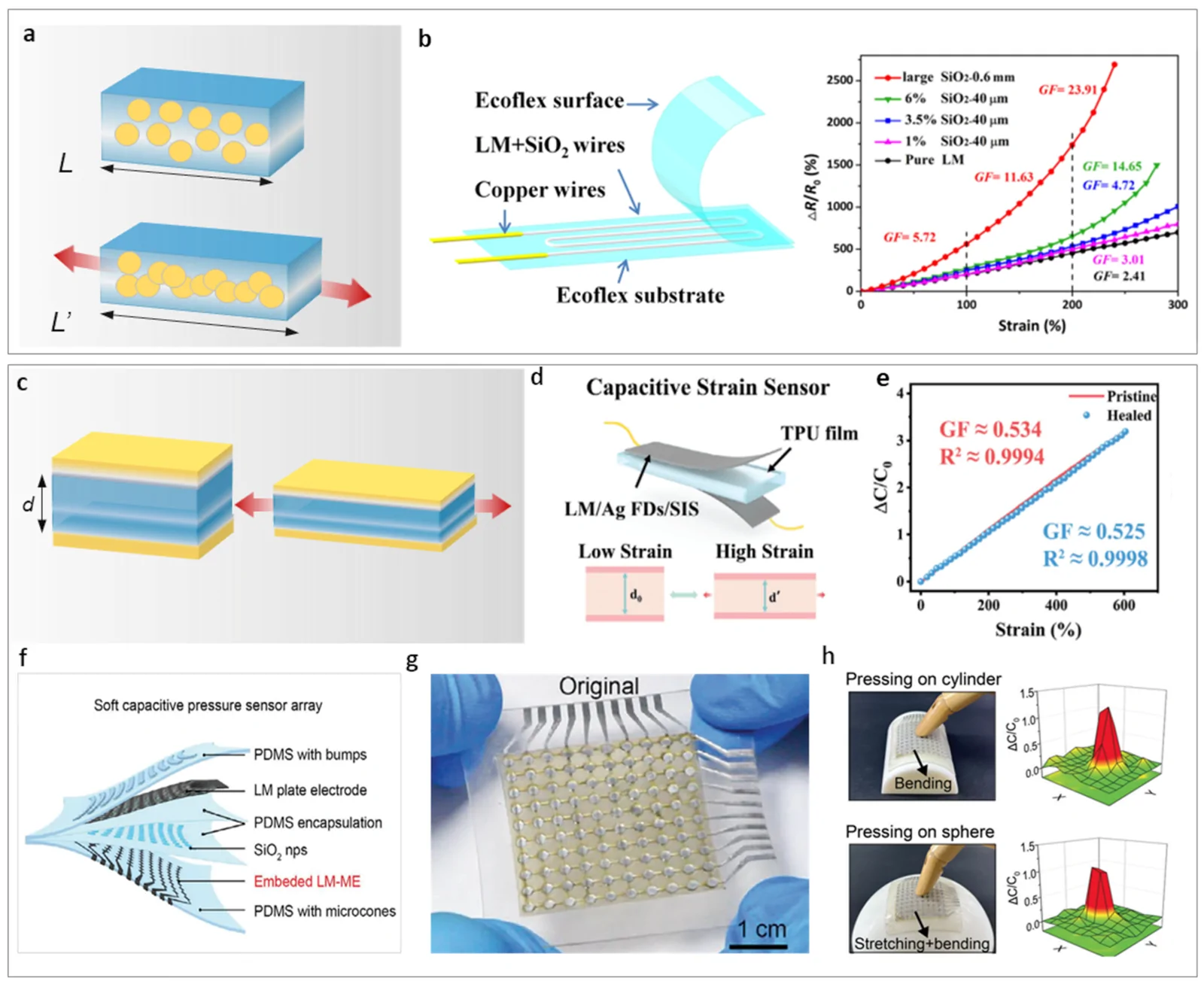
Representative liquid metal-based resistive and capacitive strain/pressure sensors. (**a**) Resistive sensing mechanism. (**b**) Printed liquid metal strain sensor and electromechanical response. (**c**) Capacitive sensing principle. (**d**,**e**) Printed self-healing capacitive sensor and strain response. (**f**–**h**) Embedded liquid metal microstructured pressure sensor array and curved surface mapping. Panels (**a**,**c**) were prepared by the authors. Panels (**b**,**d**–**h**) were reproduced with permission from Springer Nature (2023) [105], Wiley-VCH (2024) [106], and Wiley-VCH (2025) [38], respectively.

Assuming approximately constant resistivity and volume conservation during uniaxial stretching, elongation simultaneously increases L and decreases A, leading to *R*/*R*_0_ ≈ (1 + ε)^2^. Thus, the resistance change in a continuous liquid metal conductor is predominantly geometry-driven. In liquid metal composites or particulate networks, however, strain can additionally alter particle contacts, conductive junctions, and local percolation pathways, producing a substantially enhanced or nonlinear electrical response. Figure 7a schematically summarizes these two strain-sensing mechanisms.

Figure 7b shows a printed liquid metal/SiO_2_ composite strain sensor developed for wearable sensing and boxing training recognition [105]. With 0.6 mm SiO_2_ particles, the reported gauge factor increases from 5.72 at 0–100% strain to 11.63 at 100–200% and 23.91 at 200–300%; the minimum detectable strain and response time were reported as 0.05% and 158 ms, respectively. The same study reported stable response over 100 stretching–releasing cycles at 100% strain and a hysteresis value of 1.4% at 300% strain.

For capacitive sensing, liquid metal primarily serves as a compliant electrode. In the ideal parallel plate approximation:

(2) $$C=\frac{\varepsilon_{0}\cdot \varepsilon_{r}\cdot A}{d}$$

where *A* is the effective overlap area between the electrodes, *d* is the electrode separation, *ε*_0_ is the vacuum permittivity, and *ε_r_* is the relative permittivity of the dielectric layer.

According to Equation (2), the capacitance increases with increasing electrode overlap area or dielectric permittivity, whereas it decreases with increasing electrode separation. Under mechanical deformation, the soft liquid metal electrodes can undergo large geometric changes without fracture, thereby altering A and d and, in some device architectures, the effective dielectric environment. These deformation-induced changes are converted into measurable capacitance variations. Therefore, the sensing response is governed not only by the intrinsic conductivity of the liquid metal but also by the electrode geometry, dielectric properties, and deformation mode of the device.

Double helix liquid metal fibers and related architectures demonstrate how electrode geometry can convert torsion, axial strain, and touch into distinct capacitive responses [102]. Liquid metal fluidity helps maintain electrode continuity, but sensitivity remains principally determined by device geometry, dielectric mechanics, and electrode conformity rather than by an intrinsic liquid metal piezocapacitive effect.

Real devices depart from the ideal capacitor because of fringing fields, nonuniform deformation, viscoelasticity, interfacial slip, and time-dependent dielectric recovery. These effects should be separated from changes associated with the liquid metal electrode when hysteresis and long-term stability are assessed.

Figure 7d,e show a printed self-healing stretchable capacitive sensor based on a heterogeneous multilayer architecture [106]. The original study reports a highly linear capacitive response (R^2^ ≈ 0.9994) over 0–200% strain. This example demonstrates the value of modulus and interface engineering, but comparisons with resistive sensors should remain mechanism-specific because gauge factor and capacitive sensitivity are not equivalent metrics.

Figure 7f–h show an embedded liquid metal microstructured electrode array for pressure mapping on curved and deformable surfaces [38]. The embedded architecture isolates neighboring sensing elements while preserving conformability on cylindrical and spherical surfaces, illustrating how electrode geometry and mechanical support can be used to suppress crosstalk and maintain spatial mapping during deformation.

Recent work increasingly combines sensing performance with system-level functions such as gait monitoring, recyclable fabrication, signal decoding, and human–machine interfacing [104,107]. These additions are valuable, but comparisons should report device-to-device variation, calibration drift, cross-sensitivity, skin/electrode impedance where relevant, packaging state, mechanical preconditioning, and post-use recoverability alongside peak sensitivity or stretchability. Liquid metal sensors offer unusually large deformation windows, yet application relevance depends less on record sensitivity than on calibration stability, hysteresis, cross-axis interference, pixel-to-pixel variation, environmental drift, and reproducible packaging under the exact loading mode of interest.

### 4.3. Biomedical Applications

**Figure 8:**
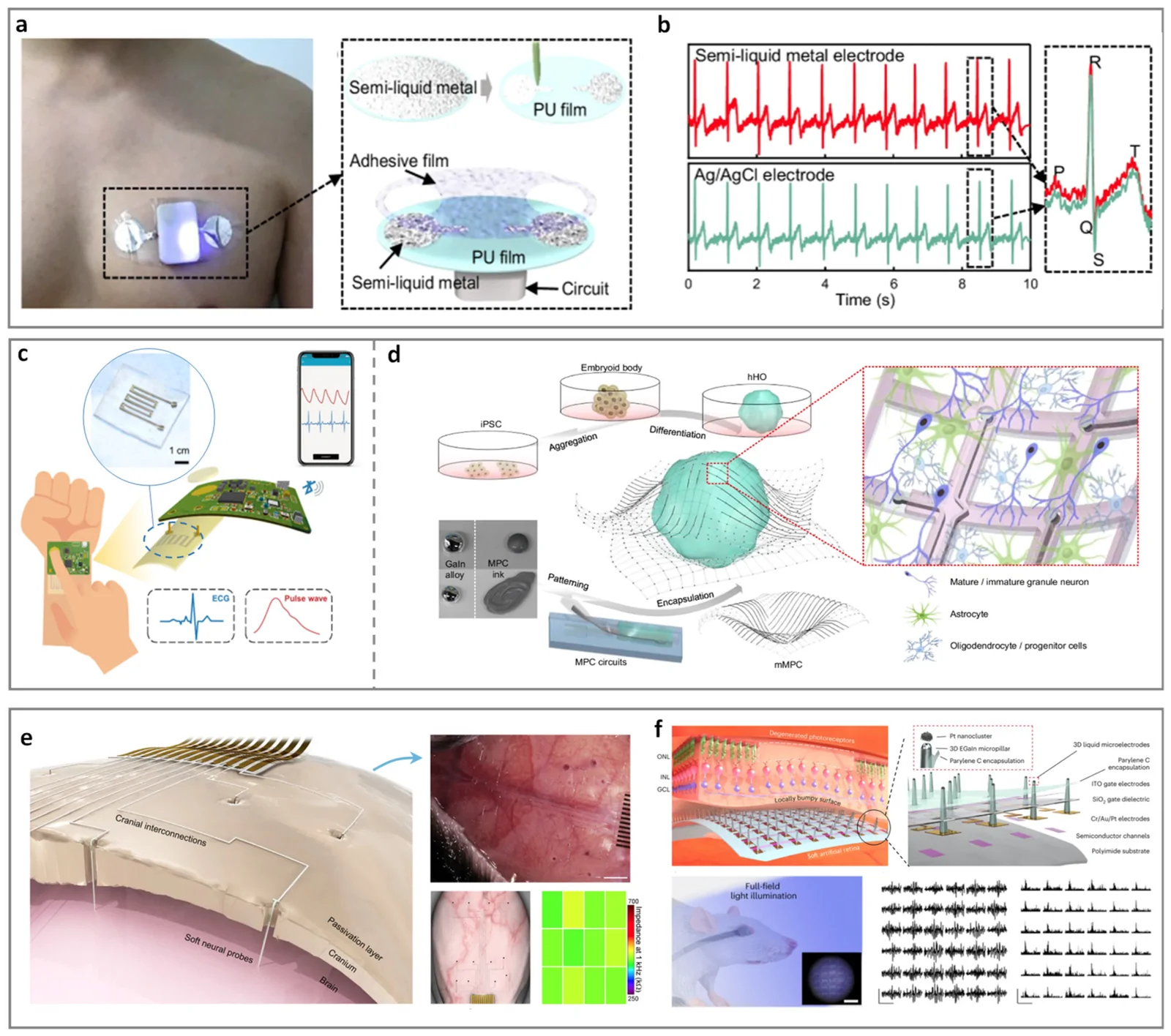
Representative biomedical applications of liquid metal electrodes. (**a**,**b**) Recyclable semi-liquid metal circuits and wearable physiological recording. (**c**) Dual-mode electrophysiological/pressure sensing for cuffless blood pressure monitoring. (**d**,**e**) Three-dimensional liquid metal neuro-interfaces for human hippocampal organoids. (**f**) Liquid metal microelectrode array integrated with an ultrathin retinal prosthesis. Panels (**a**–**f**) were reproduced with permission from Springer Nature (2025) [109], the Royal Society of Chemistry (2024) [110], Springer Nature (2024) [111], and Springer Nature (2024) [37], respectively.

Liquid metal biomedical electrodes span wearable physiological interfaces, microfluidic neural stimulation electrodes, biointegrated recording platforms, and implantable stimulation systems. Pre-aligned liquid metal electrodes have long been integrated into microfluidic platforms for neural stimulation [108], while multifunctional wearable systems combine liquid metal with other conductive materials for motion and physiological monitoring [104]. Figure 8a,b show a semi-liquid metal circuit platform that combines high-precision patterning, substrate compatibility, stretchability, reuse, and recycling, together with wearable physiological recording demonstrations [109]. The reported minimum feature scale of 5 μm illustrates how manufacturing precision and end-of-life considerations can increasingly be incorporated into bioelectronic electrode design.

Figure 8c shows a dual-mode wearable device that combines electrophysiological and pressure sensing for cuffless blood pressure monitoring [110]. Moving beyond epidermal interfaces, Figure 8d,e show a three-dimensional liquid metal/polymer neuro-interface designed for human hippocampal organoids [111]. The mesh platform provides 128 channels distributed around the organoid and is reported to remain electrically functional under large deformation, enabling higher-throughput recording from a three-dimensional neural model.

For active neural stimulation, Figure 8f shows a liquid metal-based three-dimensional microelectrode array integrated with an ultrathin retinal prosthesis [37]. Such systems illustrate the mechanical advantage of compliant conductors at delicate tissue interfaces, but implantable performance cannot be inferred from softness alone. Chronic electrical stability, hermetic packaging, sterilization, biofouling, tissue response, surgical handling, and explant/replacement procedures must be evaluated together.

LMEs are progressing from short-term wearable demonstrations toward three-dimensional and implantable interfaces, but clinical relevance will require chronic in vivo evidence, standardized electrochemical ageing, sterilization compatibility, packaging integrity, tissue response assessment, and comparison with established clinical electrodes.

### 4.4. Energy Storage and Conversion Devices

Energy-related LMEs perform fundamentally different functions depending on the device: they may act as flowable electrochemically active electrodes, deformable battery electrodes/current collectors, or highly compliant electrodes in mechanical energy harvesters. Soft EGaIn–MnO_2_ batteries and room-temperature liquid metal–air batteries demonstrate how liquid metal phases can support mechanically adaptive electrochemical architectures [112,113], whereas triboelectric systems use the liquid metal primarily as a deformable conductor. These roles require different performance metrics and should not be compared using a single conductivity or stretchability benchmark.

**Figure 9:**
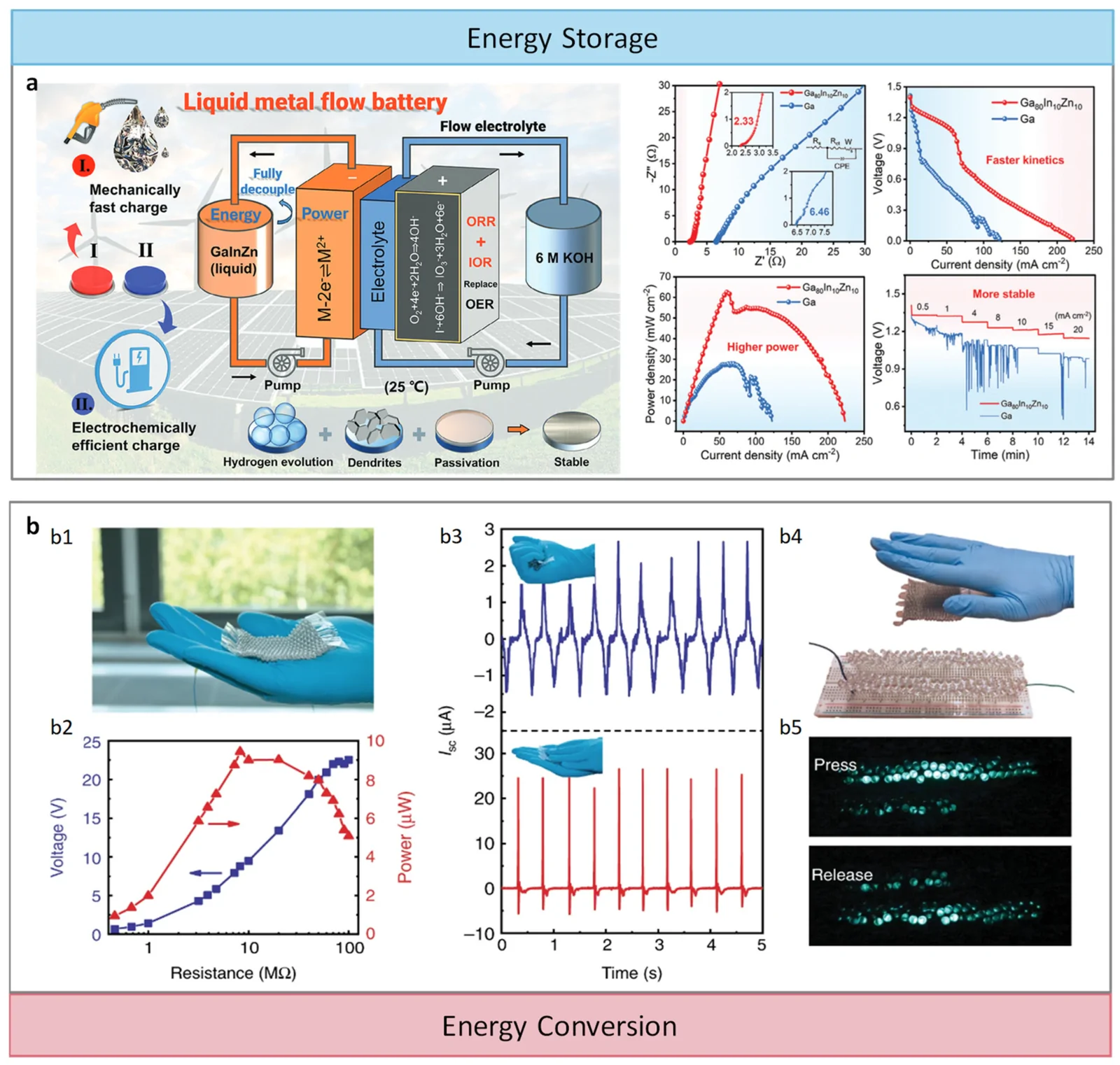
Representative energy-related liquid metal electrodes. (**a**) Room-temperature liquid metal flow battery architecture and electrochemical performance. (**b1**) Photograph of the wearable liquid metal-based triboelectric textile integrated on a glove. (**b2**) Output voltage and power characteristics under different external resistances. (**b3**) Electrical output signals generated by cyclic mechanical deformation of the triboelectric fiber textile. (**b4**) Demonstration of mechanical energy harvesting from liquid metal-based triboelectric textiles. (**b5**) Electroluminescent response driven by pressing and releasing of the triboelectric textile. Panels (**a**,**b**) were reproduced with permission from Wiley-VCH (2025) [39] and Springer Nature (2020) [114], respectively.

In electrochemical energy storage, the fluidity of liquid metals can be exploited not only to accommodate deformation but also to redefine how an active electrode is supplied and regenerated. As shown in Figure 9a, He et al. developed a room-temperature liquid metal flow battery based on a flowable Ga_80_In_10_Zn_10_ (wt.%) anode, an alkaline electrolyte, and an air electrode [39]. By combining continuous liquid metal transport with interfacial electrochemical reactions, the system supports both conventional electrochemical charging and rapid mechanical replacement of the active anode. The reported battery delivered a practical capacity of 635.1 mAh·g^−1^, while repeated discharge at 10 mA·cm^−2^ produced a total capacity of 317 mAh over 123 h with an average discharge voltage of approximately 1.1 V; mechanical refueling could be completed within 5 min. These results illustrate a distinctive advantage of flowable LMEs: electrode replenishment and electrochemical regeneration can be decoupled. Nevertheless, translation from a laboratory cell to a practical flow battery system will require simultaneous control of hydrogen evolution, corrosion, electrolyte circulation, sealing, pumping losses, stack architecture, and long-term energy efficiency.

The functional role of the liquid metal changes fundamentally when moving from electrochemical storage to mechanical energy harvesting. Rather than participating directly in redox reactions, the liquid metal can serve as a highly compliant current-collecting electrode that preserves electrical continuity during repeated textile deformation. Dong et al. demonstrated this concept using thermally drawn Geniomer fibers containing integrated Galinstan electrodes [114]. The resulting fibers retained a fracture strain of 557 ± 54%, while remaining compatible with scalable fiber processing. This mechanical compliance enables the transition from an individual fiber to a textile-scale energy harvester: Figure 9(b1) shows a 6 × 6 cm textile woven from a single continuous 4 m long, 1.1 mm thick triboelectric fiber, whereas Figure 9(b2) demonstrates the load-dependent electrical output, with the instantaneous power reaching its maximum at a load resistance of approximately 8.2 MΩ. The practical consequence of this architecture is further demonstrated in Figure 9(b3): simple hand grasping generated outputs of up to approximately 260 V and 2.5 μA, whereas the stronger tapping stimulus increased the peak output to approximately 317 V and 26 μA. These mechanically generated electrical signals were sufficient to directly illuminate an array of 100 LEDs, as shown in Figure 9(b4,b5). Thus, Figure 9b establishes a continuous progression from a deformable liquid metal electrode, through textile integration and load-matched energy harvesting, to direct electrical powering. More broadly, the comparison between Figure 9a,b highlights two fundamentally different functions of LMEs in energy technologies: the former exploits liquid metal as a mobile electrochemically active phase, whereas the latter exploits it as a mechanically compliant conductor for contact electrification/electrostatic induction-based energy conversion.

These examples emphasize that energy storage electrodes should be judged by capacity/energy metrics, Coulombic and energy efficiency, cycling, corrosion, sealing, and safety, whereas deformable energy-harvesting electrodes require conductivity under strain, charge transfer/output stability, mechanical durability, textile/process scalability, and environmental robustness. A single ‘energy application’ category, therefore, masks fundamentally different electrode functions.

Recent battery studies further extend liquid metal functions from bulk flowable electrodes to repairable, recyclable, and multifunctional electrode architectures. Liquid-gallium-based 3R batteries explicitly connect electrochemical function with resilience, repairability, and material recovery [115], while gallium–carbon composites combine printable conductive functions with integrated sensor, heater, and battery demonstrations in recyclable electronics [116]. Such systems are relevant to this review when the liquid metal directly contributes to current collection, electrochemical charge transfer, or electrode interface stabilization; otherwise, they should be treated as adjacent liquid metal materials rather than LMEs in the strict sense defined in Section 1.

Energy-related LMEs are promising because fluidity can enable either mobile active electrodes or mechanically compliant current collectors, but practical value will ultimately be determined by system efficiency, sealing and leakage control, environmental stability, manufacturability, and lifetime rather than by electrode deformability alone.

### 4.5. Technology Maturity and Translational Status

Wearable flexible electronic and sensor systems have reached integrated demonstrator and short-duration human feasibility stages, but these demonstrations should not be interpreted as evidence of manufacturing readiness. Scaling requires repeatable multilayer assembly, stable soft–rigid contacts, encapsulation, calibration retention, cleaning/washability where relevant, and statistically supported device-to-device consistency.

Biomedical implantable systems, including neural and retinal interfaces, remain predominantly preclinical or ex vivo/in vitro research platforms. Translation will require chronic biocompatibility, sterilization compatibility, hermetic packaging, stable electrode impedance and contact behavior, surgical handling, removal or replacement procedures, and regulatory evidence. Energy devices are likewise mainly laboratory demonstrators whose deployment depends on system-level sealing, environmental durability, cycling or electromechanical lifetime, and cost.

Accordingly, the maturity classification in Table 4 describes the highest validation level demonstrated in representative studies rather than assigning a formal technology-readiness level. The table separates proof of function from the additional evidence required for reproducible manufacturing, long-term operation, safety assessment, and practical or clinical deployment.

## 5. Challenges and Future Perspectives

**Figure 10:**
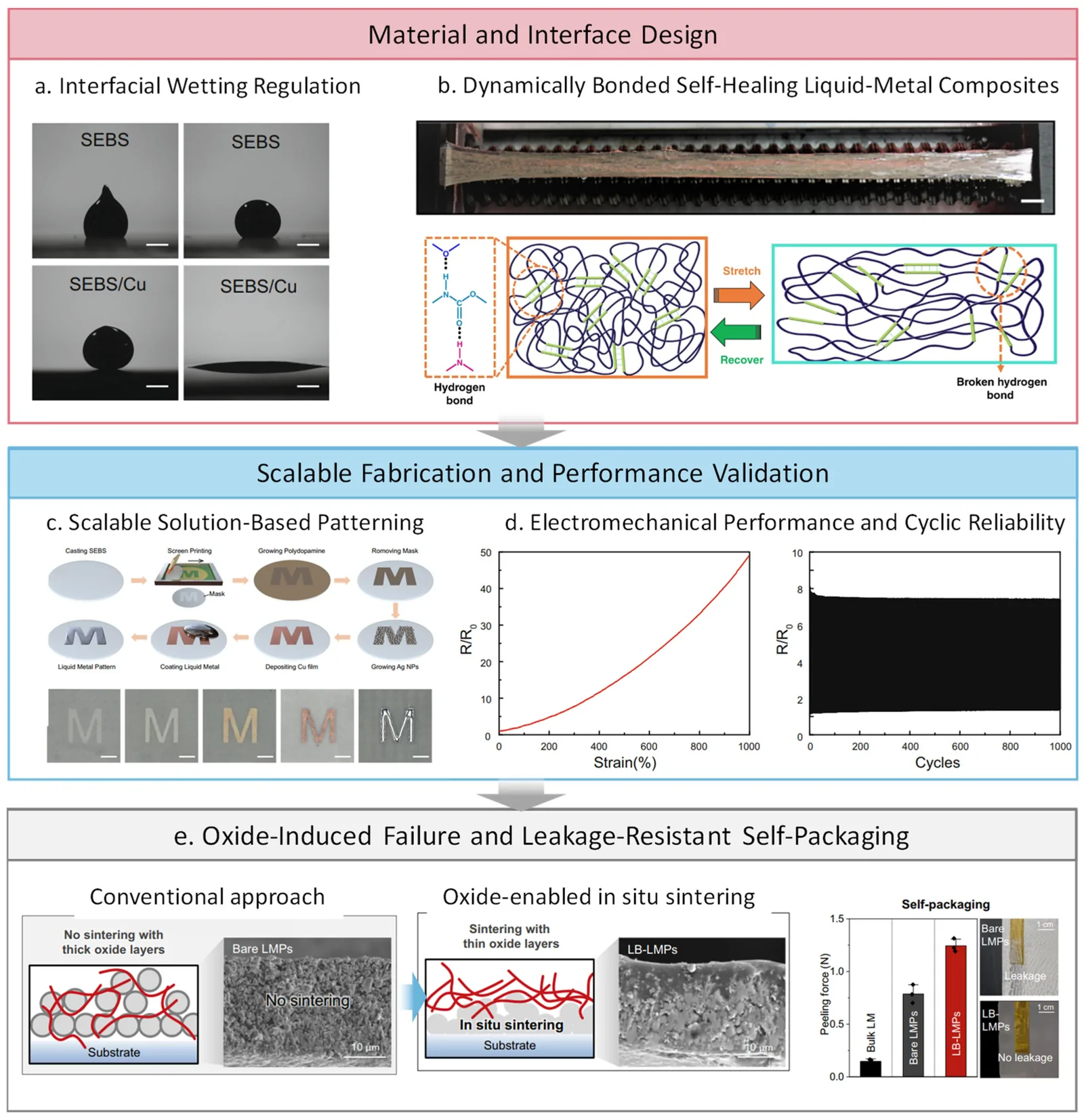
Material, fabrication, and failure mitigation strategies for reliable liquid metal electrodes. (**a**) Interfacial wetting regulation. (**b**) Stretchable and self-healing polymer–liquid metal conductor design. (**c**,**d**) Scalable solution processing and electromechanical validation. (**e**) Oxide-enabled in situ sintering and leakage-resistant self-packaging. Panels (**a**–**e**) were reproduced with permission from Springer Nature (2021) [117], Springer Nature (2019) [118], and Springer Nature (2025) [119], respectively.

Practical translation of LMEs requires simultaneous advances in interfacial control, mechanical reliability, scalable patterning, and long-term encapsulation. Figure 10a first illustrates how interfacial wetting can be deliberately engineered to define liquid metal patterns. Zhu et al. showed that Galinstan remains strongly non-wetting on SEBS after oxide removal, with a contact angle of 135.4°, whereas its contact angle on Cu decreases from 160° to only 10.7° after HCl treatment. This pronounced wettability contrast enables selective deposition on Cu-defined regions and provides a simple route toward high-fidelity patterning [117]. Building on such interface control, Figure 10b shifts the focus from pattern formation to damage tolerance. Parida et al. introduced liquid metal particles and Ag flakes into a self-healable thermoplastic elastomer, producing a conductor with an initial conductivity of 6250 S·cm^−1^ that remained conductive up to 2500% strain, with R/R_0_ of approximately 10 at the maximum strain. More importantly, 96.0% of the electrical conductivity was recovered after healing, and this recovery could be repeated over five damage–healing cycles [118].

Once interfacial selectivity and damage tolerance are established, manufacturability becomes the next critical requirement. As shown in Figure 10c, the fully solution-based process of Zhu et al. produced liquid metal features with a typical resolution of 100 μm, while coupling the process with photolithography further reduced the minimum feature size to approximately 15 μm. The patterned conductor simultaneously reached a conductivity of 4.15 × 10^6^ S·m^−1^ and a tensile strain of 1000% [117]. However, high stretchability alone does not guarantee electrical stability. Figure 10d shows that the resistance increased by approximately 1.5-, 15-, and 48-fold at 100%, 500%, and 1000% strain, respectively, although negligible resistance degradation was observed during 1000 stretch–release cycles at 300% strain. This distinction is important because maximum strain and cyclic electrical reliability represent different performance dimensions and should not be evaluated interchangeably.

Finally, Figure 10e addresses a remaining bottleneck of particle-based LMEs: the conflict between electrical activation and leakage protection. Seo et al. reduced the oxide shell thickness of ligand-bound liquid metal particles by approximately 42.8%, enabling spontaneous interparticle sintering during solvent evaporation and subsequent vertical phase separation into a liquid metal-rich conductive region and a polymer-rich packaging layer [119]. This self-packaged architecture achieved a conductivity above 8.75 × 10^6^ S·m^−1^ without additional mechanical activation, while suppressing leakage; notably, no significant oxide thickness increase was observed after 60 days, and the conductor maintained low resistance during storage. Taken together, Figure 10 demonstrates that the practical development of LMEs should not be judged by an isolated record value. Instead, interfacial wettability, conductivity, patterning resolution, strain-dependent resistance, cyclic durability, healing efficiency, leakage resistance, and environmental stability need to be considered as an integrated performance framework.

Leakage is also closely coupled with substrate contamination and environmental stability. Once encapsulation is damaged, mobile liquid metal may spread along polymer interfaces, remain as residues on adjacent surfaces, or react with metallic contacts, potentially altering adhesion, contact resistance, and device integrity. These risks become more significant during long-term exposure to humidity, elevated temperature, saline or biological media, and repeated mechanical deformation, where swelling, corrosion, interfacial degradation, or barrier layer fatigue may accelerate leakage and contamination. Therefore, future LME studies should evaluate not only whether leakage occurs, but also the chemical compatibility of adjacent materials, residual contamination after failure, barrier layer integrity, and electrical performance after standardized environmental aging.

Lifecycle design should also be treated as an experimentally measurable electrode requirement. Recent liquid-gallium batteries and gallium–carbon composites explicitly incorporate repair, recovery, or recyclable device concepts [115,116], while a binder-free EGaIn–carbon composite further demonstrates a material architecture designed for recovery and reuse [120]. These studies are important, but future sustainability claims should report recovered liquid metal fraction, purity after recovery, performance after repeated recycling, auxiliary solvent and energy inputs, and the fate of polymeric substrates or additives. Device-scale end-of-life assessment should, therefore, extend beyond whether gallium can in principle be recovered and should include disassembly, contamination, separation efficiency, and closed-loop reuse.

## 6. Conclusions

Liquid metal electrodes provide a distinctive combination of metallic conductivity, fluid deformability, and dynamically reconfigurable interfaces, yet their performance cannot be understood from bulk material properties alone. This review, therefore, examines LMEs through an electrode-centered framework that links material composition, oxide-mediated interfacial chemistry, electrode architecture, fabrication strategy, condition-dependent performance, and application requirements. Gallium-based alloys dominate room-temperature deformable electronics because of their low melting temperatures, metallic conductivity, low bulk viscosity, and oxide-assisted patternability; however, the same native oxide layer can increase contact resistance, inhibit particle sintering, and induce adhesion- or peeling-related instability, and should, therefore, be regarded as a controllable interfacial design variable rather than an intrinsically beneficial or detrimental feature. Comparison of fabrication strategies further indicates that no single approach simultaneously maximizes resolution, adhesion, deformability, throughput, scalability, and long-term durability: template-assisted patterning is suitable for repeated planar structures, direct writing offers greater geometric flexibility, microfluidic confinement improves containment and deformability, and particle-based or field-assisted methods provide additional opportunities for scalable or high-resolution fabrication but introduce activation, process window, and reproducibility constraints. Likewise, maximum strain, electrical conductivity, self-healing time, and cycle number should not be treated as isolated record values; meaningful evaluation requires simultaneous consideration of the initial material state, conductor geometry, deformation amplitude, cycling conditions, resistance drift, contact behavior, and activation mechanism. Although LMEs have progressed from fundamental material demonstrations to integrated wearable devices and preclinical bioelectronic systems, most technologies remain short of manufacturing or clinical readiness. Future advances will, therefore, require reproducible fabrication, robust soft–rigid interfaces, selective oxide regulation, leakage-resistant encapsulation, standardized electromechanical and environmental ageing protocols, application-specific safety assessment, and greater consideration of material recovery, recyclability, and end-of-life management. Establishing common reporting standards and failure-based evaluation criteria will ultimately be essential for distinguishing impressive laboratory demonstrations from liquid metal electrode technologies that can be manufactured and operated reliably in practical systems.

## Figures and Tables

**Table 2:** Comparative trade-offs and application windows of liquid metal electrode fabrication strategies.

Representative References	Typical Application Window	Principal Trade-Off	Resolution/Throughput Profile	Fabrication Strategy
[13,64,68]	Repeated planar circuits, electrode arrays, large-area sensors	Simple planar pattern definition; wetting, edge retraction, transfer residue, and alignment can limit fidelity	Moderate-to-high resolution; batch or semi-batch processing	Template- and screen-assisted patterning
[74,75,76]	Customized circuits, non-planar interconnects, rapid prototyping	High geometric freedom and conductivity; sensitive to nozzle/optics, wetting, oxide state, and line breakup	Programmable geometry; serial to projection-enabled parallel processing	Direct writing/printing of bulk or structured LM
[51,68,76,94]	Printed soft electronics, composite conductors, multimaterial devices	Improved handling and matrix integration; oxide shells/percolation can require activation and complicate comparability	Compatible with scalable printing; activation-dependent	Particle- or composite-ink printing
[90,91]	Wearable sensors, sealed interconnects, dense microfluidic electrode arrays	Excellent containment and deformability; filling defects, ports, bonding, and leakage become dominant risks	Channel-defined resolution; moderate throughput	Microfluidic/capillary confinement
[38,92,93]	Non-planar substrates, electrochemical interfaces, specialized microstructures	Localized manipulation and specialized interfaces; narrower process windows and weaker evidence of general scalability	Application-specific; equipment/process dependent	Field-, electrochemical-, and surface-assisted methods
[74,75,85]	Dense microelectrodes, miniaturized circuits, bioelectronic interfaces	High integration density; multiple steps, registration, and equipment burden	Micrometer-scale features; process-complexity dependent	High-resolution transfer/lithographic integration

**Table 3:** Condition-aware quantitative benchmarking of representative liquid metal electrode architectures.

Activation or Recovery, Stability, and Principal Limitation	Maximum Strain/Cyclic Test	Feature Size and Electrical Metric	Electrode Architecture/Fabrication	Reference
Mechanical activation required; healing and aging NR; oxide shell rupture may affect reproducibility	ε_max_ ≈ 2500%; 1500 cycles at 100% strain; terminal drift NR	≈20 μm; σ = 3.0 × 10^6^ S·m^−1^; *R*c: NR	DLP-patterned EGaIn-particle/PHEA bilayer; UV curing and mechanical sintering	[74]
No activation after LM filling; recyclability demonstrated; multistep and metal-template-dependent fabrication	ε_max_ ≈ 100%; 10,000 cycles at 100% strain; residual drift NR	19.28 μm; σ = 3.1 × 10^6^ S·m^−1^; *R*c: NR	EHD-confined EGaIn/Cu/PDMS multilayer; electrodeposition and selective wetting	[75]
No post-processing activation; aging NR; phase ratio and conductive connectivity require precise control	ε_max_ = 1200%; Δ*R*/*R*_0_ = 1.4 at 1200% strain; cycling NR	Feature size NR; σ = 2.3 × 10^6^ S·m^−1^; *R*c: NR	One-step-coated biphasic LMP/RLMP composite	[94]
Acoustic activation required; stable for ≈16 weeks in PBS; high LM loading and matrix-dependent behavior	ε_max_ ≈ 4100%; 15,000 cycles at 100%, 8000 at 300%, and 1200 at 500% strain; *R*/*R*_0_ = 1.33 at 500%	Feature size NR; σ ≈ 2.1 × 10^6^ S·m^−1^; *R*c: NR	Acoustically assembled EGaIn particle network in PU	[95]
Damage-triggered electrical recovery within microseconds; stable from −15 to 80 °C and in aqueous environments; large resistance increase at extreme strain	ε_max_ = 1200%; 1000 cycles at 300%; *R*/*R*_0_ = 38.1 at 1200%; ≈30% residual increase after release	Feature size NR; σ ≈ 1.2 × 10^6^ S·m^−1^; *R*c: NR	Screen-printed Ga–In–Sn microcapsule/Cu/SIS multilayer conductor	[96]
Damage-triggered electrical self-healing; washability demonstrated; multistep processing and healing efficiency NR	ε_max_ ≈ 1000% uniaxial and ≈525% biaxial area strain; 10,000 cycles at 500%; terminal drift NR	Feature size reported in SI; σ, *R*s, and *R*c: NR	AgNW/EGaIn microcapsule bilayer on SEBS	[10]
No post-fabrication activation; >8 months in vivo in rats; multilayer conductivity and biointerface metrics are not directly comparable with bulk conductors	ε_max_ = 1000% for 10–50 μm lines and 1500% for a 200 μm line; cyclic stability at 1000% strain	2 μm; σ = 1.3–3.9 × 10^7^ S·m^−1^ for the multilayer conductor; *R*c: NR	Wafer-patterned LM/AgIn/Ag microelectrode array on an SBS fibrous mat	[97]

Note: Electrical conductivity, sheet/line resistance, contact resistance, and impedance have different physical meanings and are not directly interchangeable. NR indicates that the corresponding value was not explicitly reported in the cited primary study.

**Table 4:** Evidence maturity and principal translational barriers of representative liquid metal electrode systems.

Principal Barriers to Practical Implementation	Representative Highest Validation	Current Evidence Level	Application Category and Representative References
Manufacturing yield, soft–rigid interface fatigue, encapsulation, component integration, abrasion/washability, repairability, and long-term human use validation	Multilayer liquid metal interconnects integrated with rigid components and deformable circuits; on-body or functional system demonstrations	Integrated wearable demonstrator	Stretchable circuits and integrated electronic skins [36,98,99]
Calibration consistency, hysteresis, cross-sensitivity, drift, device-to-device variation, packaging, and standardized aging	Strain sensing, self-healing capacitive sensing, and pressure mapping on curved/deformable surfaces	Laboratory device/wearable prototype	Flexible mechanical sensors and electronic skins [38,105,106]
Larger cohorts, clinical reference comparison, wearing comfort, skin interface stability, wireless integration, reproducibility, and regulatory evaluation	Wearable physiological recording and multimodal electrophysiological/pressure sensing	Human feasibility/wearable demonstration	Non-invasive physiological interfaces [109,110]
Chronic packaging, sterilization, biofouling, tissue response, long-term impedance stability, surgical handling, explant/replacement, and regulatory approval	High-channel-count organoid recording and retinal/neural stimulation platforms with compliant LM electrodes	In vitro/ex vivo advanced model to preclinical animal demonstration	Biointegrated/implantable neural interfaces [37,97,111]
Electrolyte management, gas evolution, corrosion, sealing, stack scale-up, system efficiency, cost, safety, and end-of-life management	Flowable Ga–In–Zn anode with mechanical and electrochemical recharging concepts	Laboratory cell proof of concept	Room-temperature liquid metal flow batteries [39]
Long-term electromechanical durability, environmental ageing, textile integration, scalable termination/interconnects, power management, and manufacturing consistency	Thermally drawn super-elastic fibers/textiles with integrated liquid metal electrodes and mechanical-to-electrical conversion	Laboratory energy-harvesting demonstrator	Liquid metal triboelectric fibers/textiles [114]

## Data Availability

No new data were created or analyzed in this study. Data sharing is not applicable to this article.
